# New insights into the role of empagliflozin on diabetic renal tubular lipid accumulation

**DOI:** 10.1186/s13098-022-00886-x

**Published:** 2022-08-23

**Authors:** Hong Sun, Juan Chen, Yulin Hua, Yuyang Zhang, Zheng Liu

**Affiliations:** 1 Department of Endocrinology and Metabolism, Dushu Lake Hospital Affiliated to Soochow University, Medical Center of Soochow University, Suzhou, Jiangsu China; 2 Department of Endocrinology, Jiangsu Province Hospital of Chinese Medicine, Affiliated Hospital of Nanjing University of Chinese Medicine, Nanjing, China; 3 The First Clinical Medical College, Soochow University, Suzhou, Jiangsu China; 4 Department of Endocrinology and Metabolism, The First Affiliated Hospital of Soochow University, Suzhou, Jiangsu China

**Keywords:** Diabetic kidney disease, Advanced glycation end products, Empagliflozin, Renal fat fractions

## Abstract

**Background:**

Glucose cotransporter (SGLT) 2 suppression provides potent renal protective effect during diabetic kidney disease (DKD). This work aimed to explore how empagliflozin (EMPA, the selective and strong inhibitor of SGLT2) affected renal lipid deposition among patients undergoing type 2 diabetes mellitus (T2DM), a T2DM mouse model and human renal proximal tubular epithelial (HK-2) cells.

**Methods:**

This work divided subjects as 3 groups: non-diabetic volunteers, patients treated with metformin and those treated with metformin plus EMPA. In an in vivo study, EMPA was adopted for treating db/db mice that were raised with the basal diet or the high-advanced glycation end products (AGEs) diet. In addition, AGEs and/or EMPA was utilized to treat HK-2 cells in vitro.

**Results:**

Results showed that diabetic patients treated with metformin plus EMPA had lower AGEs levels and renal fat fraction (RFF) than those treated with metformin. Moreover, a significant and positive association was found between AGEs and RFF. Results from the basic study showed that EMPA decreased cholesterol level, tubular lipid droplets, and protein levels related to cholesterol metabolism in AGEs-mediated HK-2 cells, kidneys of db/db mice and those fed with the high-AGEs diet. Additionally, EMPA decreased AGEs levels in serum while inhibiting the expression of receptor of AGEs (RAGE) in vitro and in vivo.

**Conclusion:**

EMPA inhibited the AGEs-RAGE pathway, thereby alleviating diabetic renal tubular cholesterol accumulation.

## Introduction

Diabetic kidney disease (DKD) refers to the diabetes-induced chronic kidney disease (CKD) that affects around 40% of diabetic patients [1]. DKD is a global health burden and even though it has been studied for years, its molecular mechanism remains largely unclear. The factors affecting DKD are usually diabetes-related metabolic changes that induce renal pathological alterations and result in impair kidney functions. One of the critical metabolic changes that causes DKD is nonenzymatic glycosylation (NEG). NEG process irreversibly attaches the reducing sugar onto the free amino group in proteins. Amadori products and Schiff base can be produced during the above process, eventually generating the advanced glycation end products (AGEs) [2]. Serum AGEs contents among diabetics having renal insufficiency are abnormally high. There is progressive structural damage to the kidney, caused by AGEs, resulting in impairment of renal functions in DKD patients [3].

By integrative analyses of different studies, we discovered that a complete description of the typical DKD histological changes, which mainly emphasizes nodular and diffuse glomerulosclerosis, is lacking. Nonetheless, tubulointerstitial changes play a critical role in renal insufficiency in DKD cases when compared with glomerulopathy [4, 5]. AGEs cause tubular damage that progress to DKD. Excess AGEs content leads to oxidative stress [6], inflammation [7], or apoptosis [8] in renal tubules. In the previous study, we had shown the role of AGEs in inducing cholesterol production that is mediated by 3-hydroxy-3-methylglutaryl coenzyme A reductase (HMGCoAR) and cholesterol absorption mediated by low-density lipoprotein receptor (LDLr) [9–11]. When excessive cholesterol is successfully absorbed and synthesized, tubular foam cells are produced in the kidneys. Sterol regulatory element-binding protein-2 (SREBP-2), along with chaperone SREBP cleavage-activating protein (SCAP), has an important effect in modulating HMGCoAR and LDLr [12]. Once cholesterol is required in cells, SCAP can deliver SREBP-2 into Golgi from the endoplasmic reticulum (ER) and activates the latter via proteolytic cleavage. In addition, nuclear translocation of N-terminal in SREBP-2 (nSREBP-2) is attained once cleaved and processed. Thus, nSREBP-2 can activate HMGCoAR and LDLr within the cell nucleus, promoting cholesterol absorption and production. However, if cells do not require cholesterol, the SCAP-SREBP-2 complexes stay within ER, which then down-regulates the expression of HMGCoAR and LDLR. Such SCAP-mediated feedback regulation prevents cholesterol overload within cells under homeostatic conditions [13]. While, this regulation is disrupted under diseased conditions like type 2 diabetes mellitus (T2DM) [11, 14].

Progress in science and technology has aided the development of novel antidiabetic agents. Recently, the glucose cotransporter (SGLT) 2 inhibitor has shown high efficacy if a specific treatment protocol is followed [15]. Being located in apical proximal tubular cells, SGLT2 is responsible for 90% of renal glucose reabsorption [16]. SGLT2 inhibitor mainly promotes glucose excretion and decreases the blood glucose level. Randomized clinical trials (RCTs) have reported such inhibitors to be an unexpected benefit on renal outcomes, without regard to their glycemic regulation effect [17, 18]. SGLT2 inhibitors can directly alleviate renal damage by inhibiting tubular injury-associated signaling pathways related to inflammation or oxidative stress [19–21]. Wang and colleagues discovered the SGLT2 inhibitor that regulated lipid metabolism in mouse kidney and prevent DKD progression [22]; the underlying mechanism was unspecified. This study aims to elucidate mechanisms underlying SGLT2’s selective inhibitor EMPA, which improves the lipid deposition within the kidney of T2DM patients, a high-AGEs diet-fed T2DM mouse model, as well as AGEs-exposed HK-2 cells.

## Methods

### Subjects and study design

We recruited patients with T2DM at the Department of Endocrinology from The First Affiliated Hospital of Soochow University. Patients who conforming to the following criteria were included: (1) patients between the age of 18–75 years; (2) patients who could undertake magnetic resonance imaging (MRI); (3) those who consented to participate in the present study; (4) patients under treatment with metformin or metformin combined with EMPA for at least 3 months. The patients with the following criteria were excluded: (1) those whose body mass index (BMI) < 18 kg/m^2^; (2) those with unstable physiological conditions or serious psychiatric disorder; (3) pregnant women.

This prospective work was approved by Local Ethics Committee of our hospital (Ethical No. 145). Each participant provided written informed consent before the examinations. We recruited 70 patients between November 2019 and October 2021. We eliminated patients who could not hold their breath in MRI examination (n = 2) or those who quit (n = 2). Finally, we enrolled 66 participants for data analyses. Among 66 subjects, 15 were normal volunteers with no history of diabetes and no abnormality showed up in the laboratory tests concerning serum creatinine (Scr), glycated hemoglobin (HbA1c), and fasting blood glucose (FBG) level. The rest 51 T2DM patients were assigned into two groups: those treated by metformin (n = 31, T2DM group) and those treated with a combination of metformin and EMPA (n = 20, T2DM + EMPA group).

### Anthropometric and biochemical measurements

Patients were physically examined, including their heights and weights and BMI [weight (kg) divided by the square of height (m^2^)]. We also collected peripheral blood samples (in a fasting state) from every patient to analyze FPG, SCR, total cholesterol (TC), and total triglycerides (TG) with the automated biochemical analyzer (HITACHI 7600, HITACHI Company, Japan). We determined HbA1c level by the automated glycosylated hemoglobin analyzer (HLC-723G8, TOSOH) and human serum AGEs contents by the ELISA kit per specific protocols (CUSABIO, Wuhan, China).

### Dixon MRI for renal fat measurements

The subjects underwent an MRI scan about 1 h after lunch. Each subject was asked to lie in the supine position during MRI scanning, with the 8-channel receiver coil being adopted for the concentration of standard torso phased-array coil in the liver at 1.5 T (SuperVan, Lonwin Medical System, China). Scanning protocols included the original localizer images and subsequent axial images by adopting the multi-echo liver-interpolated volume-excitation sequence using parameters below including 3 echoes of 2.25, 3.37, and 4.5 ms, separately, one 12° flip angle, forty 2.5 mm slices, a 256 × 205 mm matrix, a 400 × 320 mm field of view, and 38-s overall acquisition time (the initial 19-s scanning under free-breathing state, then 19-s scanning under breath-holding state). The participants held the breath in their final inspiration for ensuring data consistency. We adopted a plug-in algorithm to automatically generate MRI-FF maps using WinStation software (WinStation, Lonwin Medical System, China). An experienced radiologist, kept unaware of the study design to avoid any bias, reviewed the images by WinStation. We chose three central slices from bilateral renal sides. Thereafter, we manually put regions of interest (ROIs) onto every slice within the whole renal parenchyma. We determined the average renal fat fraction (RFF) by taking the average value of bilateral side measurements for every participant. The coefficient variations (CV) of repetition is 4%.

### Animal experiments

The study with db/db (diabetic) as well as db/m (non-diabetic) male mice of strain C57BL/KsJ was conducted at National Model Animal Centre of Nanjing University (Nanjing, China). This work followed the updated Helsinki Manifesto. The Ethics Committee of Soochow University approved our study protocols. All mice were kept in polypropylene cages at 22 ± 2 °C, 60 ± 5% relative humidity (RH) and 12-h:12-h light/dark cycle. Animals were raised for 2 weeks. Specifically, we raised a group of 8-week-old db/db mice with the AGE-rich diet, and the other group was fed with AIN-76 basal diet (Xietong Biology Co., Ltd., Nanjing, China). AIN-76 basal diet contains 64% carbohydrate, 20% protein, 3.9 Kcal/g energy, and 7% fat; following 10-min heat treatment under 90 °C, diet-derived AGEs were produced from the AIN-76 basal diet [23]. We then classified animals into five groups: including Group 1 (db/m mice, control) raised on AIN-76 basal diet; Group 2 (db/db mice) raised on AIN-76 basal diet; Group 3 (db/db mice + EMPA) raised on AIN-76 basal diet plus intragastric administration of EMPA (10 mg/kg, MedChemExpress, China) once a day from week 6 to week 8; Group 4 (db/db mice + AGEs) raised on an AGE-rich diet; Group 5 (db/db + AGEs + EMPA) raised on an AGE-rich diet plus intragastric administration of EMPA (similar to Group 3). We raised mice in respective metabolic cages and collected their urine at 24-h. The week eight samples were used for subsequent analyses. Blood samples were subjected to biochemical tests, while renal tissue samples were processed for histological analysis.

### Biochemical assays

Each mouse was sacrificed after the experiments were over. We collected blood samples from the right ventricle of the heart for biomechanical analyses performed later. We measured the levels of blood urea nitrogen (BUN), SCR, FBG, TG, and TC by a fully-automated biochemical analyzer (Hitachi). The urinary neutrophil gelatinase-associated lipocalin (u-NGAL, the marker for renal tubular injury) as well as serum AGEs were measured with an ELISA kit (CUSABIO; Wuhan, China). Meanwhile, to detect protein content from the urine collected at 24-h, this work conducted Coomassie brilliant blue protein assay (Jiancheng Bioengineering Institute, Nanjing, Jiangsu).

### Renal morphology

The renal cortex was embedded in paraffin and stored appropriately. The 3-µm cross-sections of tissue were kept on the gelatin-coated slides, followed by hematoxylin-eosin (HE) as well as periodic acid Schiff (PAS) staining.

### Cell culture

This work obtained HK-2 cells in American Type Culture Collection (Manassas, VA, USA), then cultivated them following the method described elsewhere [11]. All assays were conducted within serum-free RPMI 1640 medium containing 0.2% bovine serum albumin (BSA, fatty acid free; SigmaAldrich; Merck KGaA). We obtained EMPA in MedChemExpress (Shanghai, China) and AGEs-BSA from Abcam (Cambridge, UK). HK-2 cells were cultivated in experimental medium including 200 µg/mL AGEs-BSA, 500 nM EMPA, or the combination of these two for 48-h.

### Lipid deposition measurement

We used oil red O stain to observe lipid deposition within db/db mouse kidneys as well as HK-2 cells. In brief, four critical steps were needed: after fixation using 4% paraformaldehyde (PFA), samples were subject to 30-min oil red O staining, 5-min HE staining, and stained slides were observed under the optical microscope (Carl Zeiss, Hertfordshire, UK).

### Intracellular cholesterol quantification

Enzymatic analysis (Applygen Technologies Inc., Beijing, China) was performed to quantify free cholesterol (FC) and TC content under both in vivo and in vitro conditions, as described earlier [9, 11]. Cholesterol ester (CE) was evaluated as per the following equation, CE = TC − FC.

### Protein isolation and Western-blot (WB) assay

The nuclear and whole-cell proteins were extracted and denatured before WB assay following the protocol described elsewhere [11, 24]. We incubated the blots with antibodies specific for SREBP-2, SCAP, receptor of AGEs (RAGE), HMGCoAR, LDLr, and GAPDH (dilution, 1:200–1000, Abcam, Cambridge, UK). Incubation was carried out in Tween 20 (TBST), supplemented with 5% BSA, overnight at 4 °C. Blots were rinsed, and membranes were further probed for 1 h using secondary antibody (1:5000, within TBST, supplemented with 1% BSA; Santa Cruz Biotechnology) under ambient temperature. Finally, blots were exposed to X-ray films for detecting bands after the use of ECL (Pierce, Rockford, IL, USA). To quantify protein expression, we assessed band density using LabWorks software (UVP Laboratory Products, Upland, CA, USA); GAPDH was applied as the internal reference.

### Confocal microscopy

As described previously, human SCAP cDNA was ligated with pEGFP-C1 vector at BstE-XbaI restriction sites (Genechem Co. Ltd., Shanghai, China) for creasing the green fluorescent protein (GFP)-SCAP expression construct [9, 11]. The clone, pEGFP-SCAP, was transfected into cells using the Effectene Transfection Reagent (Invitrogen, Paisley, UK) in line with specific protocols. Afterward, we inoculated HK-2 cells onto the chamber slides. At 48-h after diverse treatments, this work fixed cells for 30 min using 5% formalin, followed by 15-min permeabilization using 0.25% Triton X-100, and 2-h addition of Golgin-97 antibody (Molecular Probes, Inc., Eugene, OR, USA) at room temperature. Samples were then washed and incubated for 1 h with the secondary antibody. This work utilized Zeiss LSM 510 Meta confocal microscope (Carl Zeiss, Hertfordshire, UK) to visualize the slides.

### Coimmunoprecipitation (Co-IP)

Co-IP was performed to analyze SCAP-SREBP-2 interaction within HK-2 cells. We initially isolated total proteins using the cell IP lysis buffer (Thermo Scientific Pierce, USA). After sample centrifugation, this work removed cell lysates using the protein A/G-agarose beads. In addition, supernatant was collected for immunoprecipitation with appropriate antibodies. Samples were further incubated with additional antibodies for immunoblotting assay.

### Statistical analysis

Experimental data were analyzed by SPSS20.0 and presented as mean ± SD, mean ± S.E.M, or median (25th and 75th percentiles). One-way ANOVA or Bonferroni test was used for multiple data comparisons. The association of AGEs with RFF was assessed by Spearman’s correlation. p < 0.05 stood for statistical significance.

## Results

### Anthropometric and demographic features

This study enrolled 51 T2DM patients (metformin treated patients: n = 31; Metformin plus EMPA treated patients: n = 20) and 15 healthy subjects. Differences in age and gender were not significant across the three groups. T2DM group had an increased BMI, FPG, HbA1c, SCR, TC, TG, AGEs, and RFF than the non-diabetic control group. Patients receiving metformin plus EMPA showed decreased BMI, HbA1c, AGEs, and RFF compared to the patients receiving metformin alone (Table 1).

**Table 1:** Characteristics of the study population

Parameter	Non-diabetic control	T2DM	T2DM + EMPA	*P* value
Number	15	31	20	NA
Age (years)	46.53 ± 10.32	45.68 ± 11.12	41.55 ± 10.15	0.304
Sex (M/F)	7/8	19/12	12/8	0.632
BMI (kg/m^2^)	21.67 ± 2.29	27.01 ± 4.14**	24.69 ± 3.32^##^	< 0.001
FBG (mmol/L)	5.09 (4.80, 5.30)	8.06 (6.27, 10.73)**	7.69 (6.05, 8.73)	< 0.001
HbA1c (%)	5.25 ± 0.27	9.26 ± 2.36**	7.51 ± 1.27^#^	< 0.001
SCR (µmol/L)	46.27 ± 6.62	61.20 ± 14.74**	59.74 ± 11.92	0.001
TC (mmol/L)	3.87 ± 1.05	5.02 ± 1.35*	4.87 ± 1.39	0.021
TG (mmol/L)	1.62 (0.84, 2.07)	2.80 (1.51, 3.99)*	2.06 (0.88, 2.26)	0.008
AGEs (µg/mL)	3.87 ± 1.76	11.72 ± 4.27**	6.79 ± 2.59^##^	< 0.001
RFF (%)	3.14 ± 1.08	5.30 ± 0.93**	4.18 ± 1.14^#^	< 0.001

Variables with normal distribution were presented as the mean ± SD, whereas those with abnormal distribution as the median (25th, 75th percentiles). *P* values were obtained from one-way ANOVA for normally distributed variables or Kruskal–Wallis H test for skewed distributed variables. Categorical data were evaluated by the chi-square test (sex)

*P < 0.05, and **P < 0.01 vs. Non-diabetic control; ^#^*P* < 0.05, ^##^*P* < 0.01 vs. T2DM

### Correlation between RFF and AGEs

**Fig. 1:**
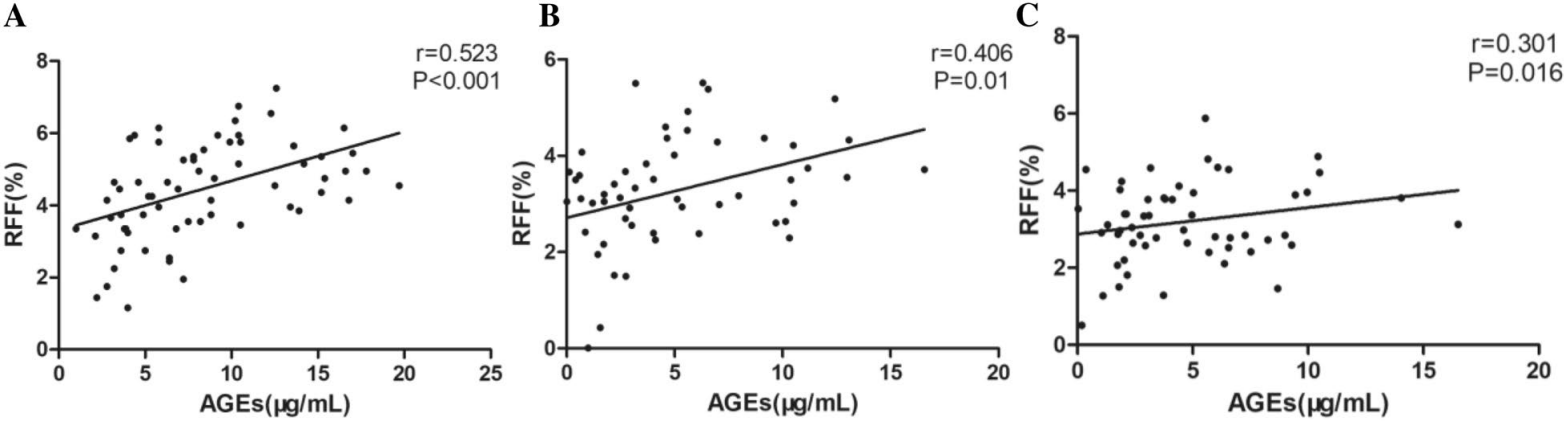
Correlation between RFF and AGEs. Association of RFF with AGEs (**A**). Associations of RFF with AGEs after adjustment for TC and TG (**B**). Associations of RFF with AGEs after adjustment for TC, TG, BMI, SCR, FBG and HbA1c (**C**)

Our finding suggests that AGEs are positively related to RFF (Fig. 1A). Even after statistically adjusting TG and TC values, RFF still showed positive relation to AGEs (Fig. 1B). After adjusting for BMI, HbA1c, FBG, and SCR, the association of RFF with AGEs were still statistically significant, albeit it had declined slightly (Fig. 1C).

### Role of EMPA in biochemical features and renal morphology in diabetic mice

**Fig. 2:**
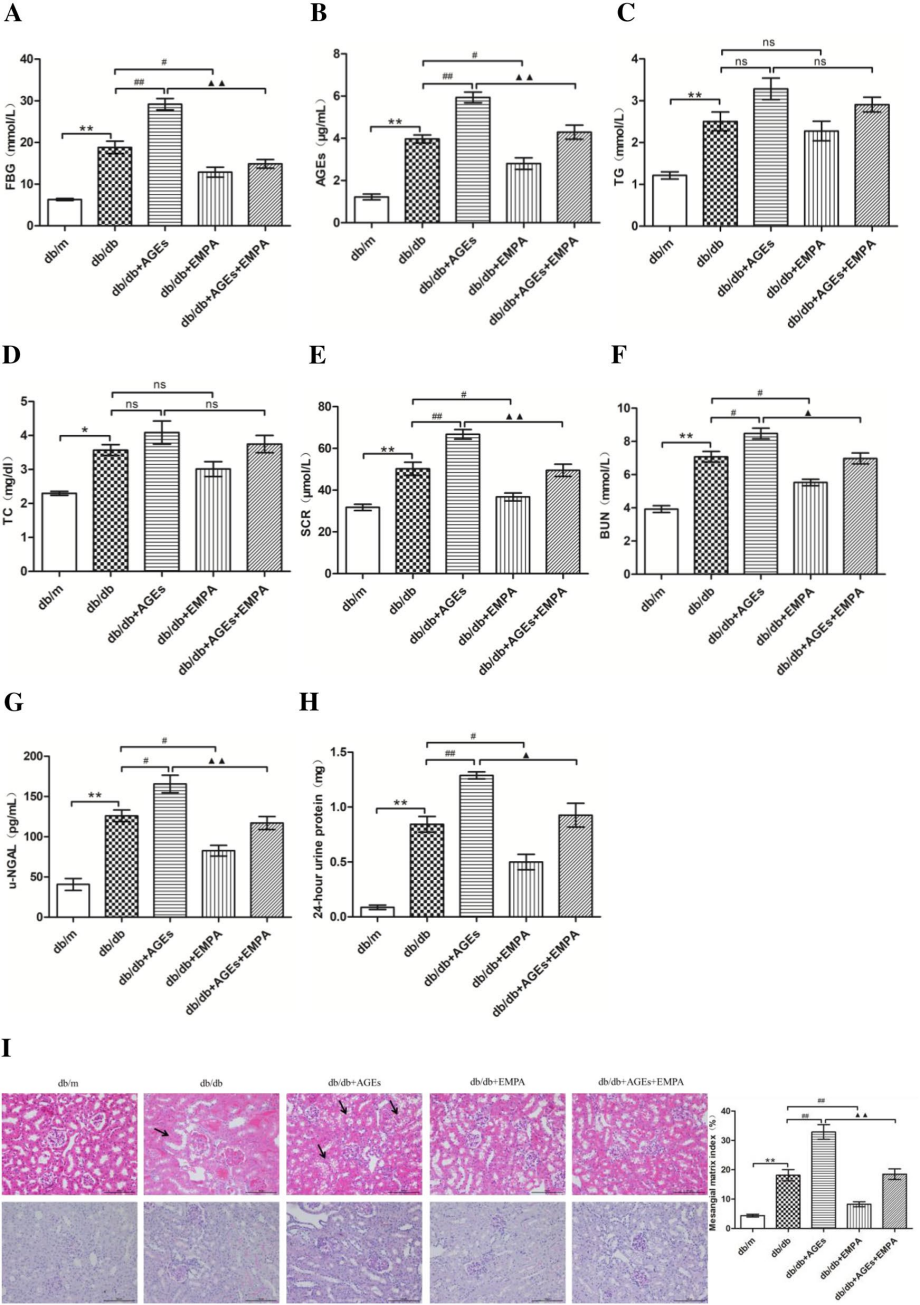
Effects of EMPA on biochemical characteristics and renal morphology of diabetic mice. The levels of FBG (**A**), AGEs (**B**), TG (**C**), TC (**D**), SCR (**E**), BUN (**F**), u-NGAL (**G**) and 24-h urine protein (**H**) in mice. HE and PAS (**I**) staining in the kidneys of mice was observed under a light microscope (200×). The mesangial area was expressed quantitatively by calculating the percentage of the total glomerular area that was PAS positive. Fifteen glomerular tufts per animal were chosen randomly for analysis. The histogram represents the mesangial matrix index. The values are expressed as the mean ± S.E.M. of 5 independent experiments. *P < 0.05 and **P < 0.01 versus the db/m group; ^#^*P* < 0.05 and ^##^*P* < 0.01 versus the db/db group; ^▲^*P* < 0.05 and ^▲▲^*P* < 0.01 versus the AGEs + db/db group; *ns *not significant

After the experiments, we acquired general mouse features (Fig. 2). The diabetic mice had high FBG levels (Fig. 2A). In comparison, db/db mice had increased AGEs (Fig. 2B), TG (Fig. 2C), TC (Fig. 2D), SCR (Fig. 2E), BUN (Fig. 2F), u-NGAL (Fig. 2G), and proteins contents in urine collected at 24-h (Fig. 2H). The AGE-rich diet-fed db/db mice showed significantly high expression the above factors (except for TC and TG values) compared with db/db mice. EMPA treatment led to marked reductions in the levels of AGEs, FBG, BUN, SCR, proteins in urine (collected at 24-h), and u-NGAL, whereas insignificant change was observed in the levels of TG or TC in db/db mice receiving the basal or an AGE-rich diets.

The HE staining did not reveal any noticeable pathological indications in the tubules or glomeruli of db/m mice. Renal tubular vacuolar degeneration could be seen in db/db mic, which significantly increased among AGE-rich diet-fed db/db mice. However, the EMPA enhanced renal tubular morphology (Fig. 2I). The PAS staining result showed that the db/m mice had a clear glomerular vascular loop and intact tubular basement membrane. However, db/db mice exhibited thickened tubular basement membrane and expanded renal glomeruli, which were conspicuous in kidneys of an AGE-rich diet-fed db/db mice. Interestingly, EMPA remarkably mitigated these pathological changes (Fig. 2I).

### Role of EMPA in in-vitro and in-vivo renal lipid deposition

**Fig. 3:**
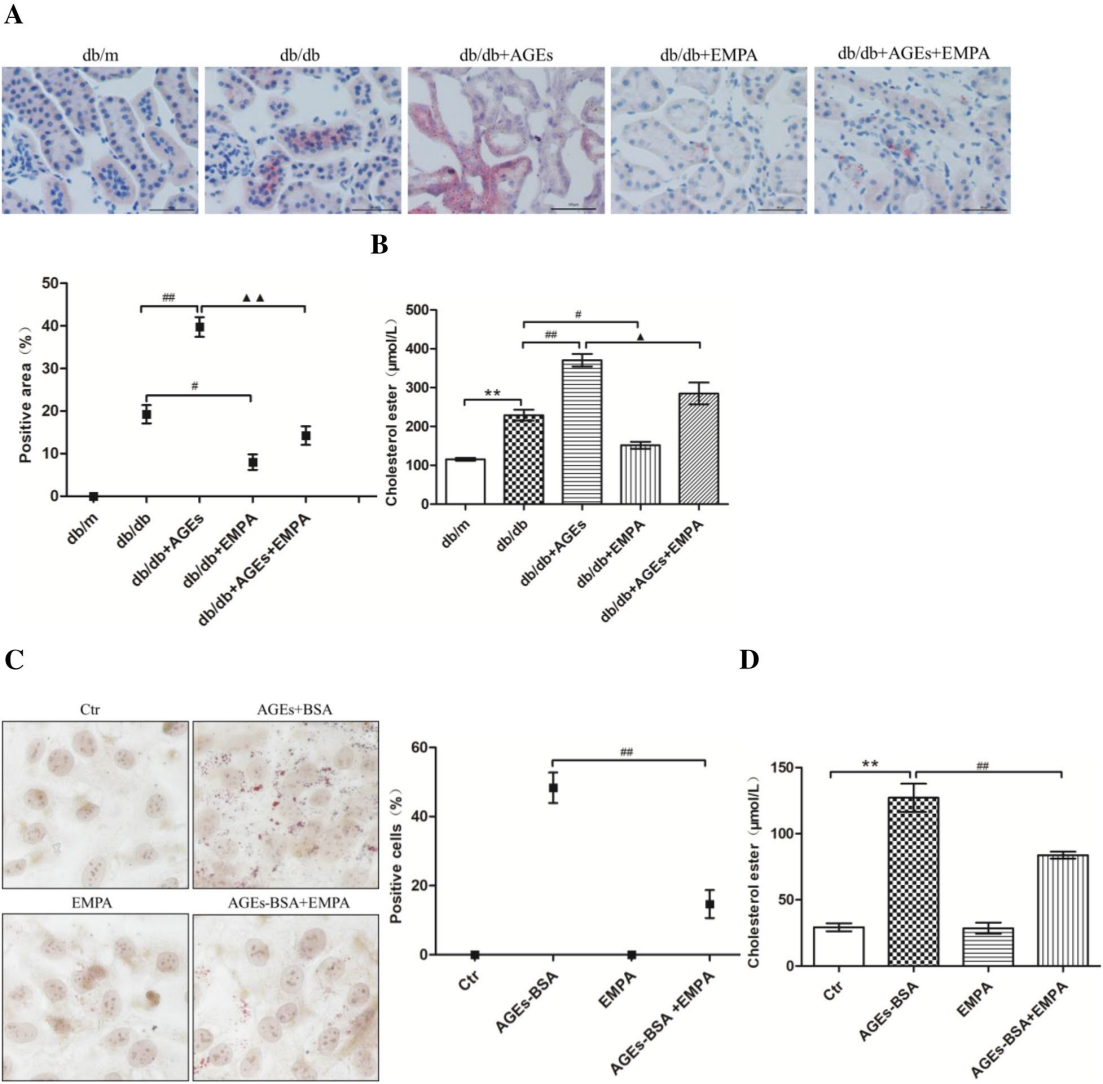
Effects of EMPA on renal lipid accumulation in vivo and in vitro. Renal oil Red O staining and the semi-quantitative analysis for the percent of the positive areas in each group (**A**). The intracellular cholesterol contents in the kidneys of mice (**B**). Oil Red O staining in the HK-2 cells and the positive percentage of HK-2 cells (**C**). The intracellular cholesterol content in HK-2 cells (**D**). Oil Red O staining was observed under a light microscope (×400). The percentage of positive staining areas in the kidneys of mice was semi-quantitative analyzed by Image J. The positive percentage of HK-2 cells was counted from 5 experiments. Values of intracellular cholesterol content are expressed as the means ± S.E.M. of 5 independent experiments. **P < 0.01 versus the db/m group or the control cells (Ctr); ^#^*P* < 0.05 and ^##^*P* < 0.01 versus db/db group or AGEs-BSA treated cells; ^▲^*P* < 0.05 and ^▲▲^*P* < 0.01 versus the AGEs + db/db group

For assessing the role of EMPA in alleviating lipid accumulation in tubular cells, lipid droplets were observed, and intracellular cholesterol levels were evaluated within db/db mouse kidneys. The db/m mouse kidneys showed no color with the oil red O staining indicating no lipid deposition, but there were strong positively-stained regions in db/db mouse renal tubules (Fig. 3A). After quantifying the intracellular cholesterol contents, it was found that cholesterol levels had enhanced within db/db mouse kidneys (Fig. 3B). Also, oil red O staining could be seen within control db/db mice; normal intracellular cholesterol levels were found within AGE-rich diet-fed db/db mice. EMPA exposure decreased the intracellular cholesterol levels as well as tubular lipid droplets within basal diet- or AGE-rich diet-fed db/db mice (Fig. 3A, B).

We then analyzed how EMPA affected lipid deposition within renal tubular cells under in vitro conditions. According to Fig. 3C, lipid droplet depositions and cholesterol levels (Fig. 3D) were significantly increased within HK-2 cells exposed to AGEs-BSA treatment. Nevertheless, using EMPA decreased both lipid droplets and intracellular cholesterol levels within AGEs-BSA-exposed HK-2 cells (Fig. 3C, D).

### Role of EMPA in the in-vivo and in-vitro metabolism of renal cholesterol

**Fig. 4:**
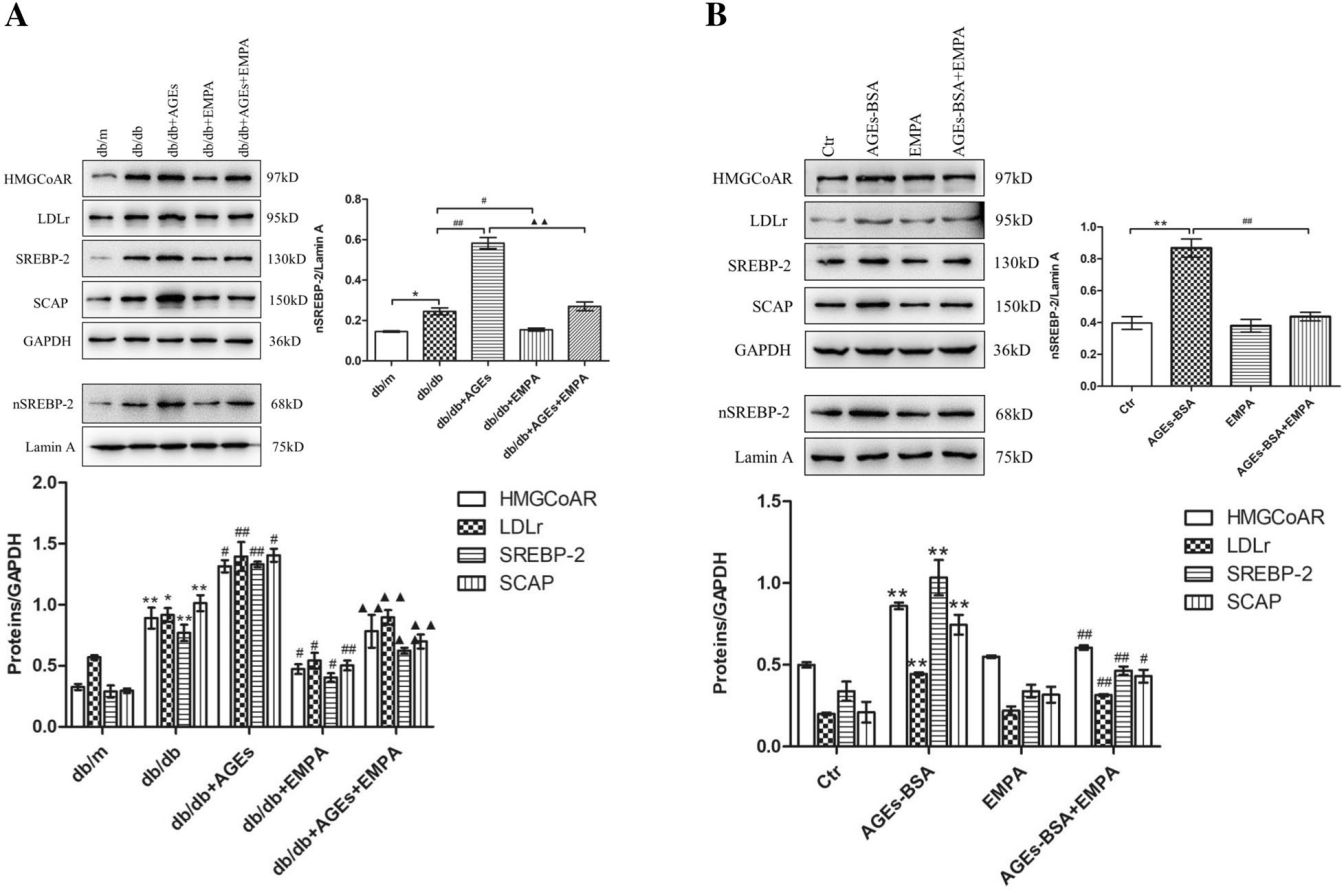
Effects of EMPA on HMGCoAR, LDLr, SREBP-2, nSREBP-2 and SCAP expression in the kidneys of diabetic mice and HK-2 cells. The protein expression of HMGCoAR, LDLr, SREBP-2, nSREBP-2 and SCAP in the kidneys of mice (**A**) and in HK-2 cells (**B**) was determined by Western blotting. Image J was used to quantify the relative levels of proteins. GAPDH or Lamin A was used as an internal control. The values are expressed as the mean ± S.E.M. of 5 independent experiments. *P < 0.05 and **P < 0.01 versus the db/m group or the control cells (Ctr); ^#^*P* < 0.05 and ^##^*P* < 0.01, versus db/db group or AGEs-BSA treated cells; ^▲▲^*P* < 0.01 versus the AGEs + db/db group

To investigate how EMPA affected cholesterol metabolism, this study measured LDLr, HMGCoAR, SCAP, SREBP-2, and nSREBP-2 levels within diabetic mouse kidneys; the result shows elevated levels within db/db mouse kidneys (Fig. 4A) relative to control. AGE-rich diet-fed db/db mice had obviously higher LDLr, SCAP, HMGCoAR, SREBP-2 and nSREBP-2 protein expression in comparison with the db/db mice. But EMPA-exposed db/db mice, fed with either basal diet or diet rich in AGEs, had markedly reduced protein levels.

We also analyzed how EMPA affected HMGCoAR, LDLr, SCAP, nSREBP-2, and SREBP-2 expression in HK-2 cells. Protein levels significantly increased within AGEs-BSA-exposed HK-2 cells (Fig. 4B) compared to the control, while EMPA exposure inhibited their high expressions in HK-2 cells.

**Fig. 5:**
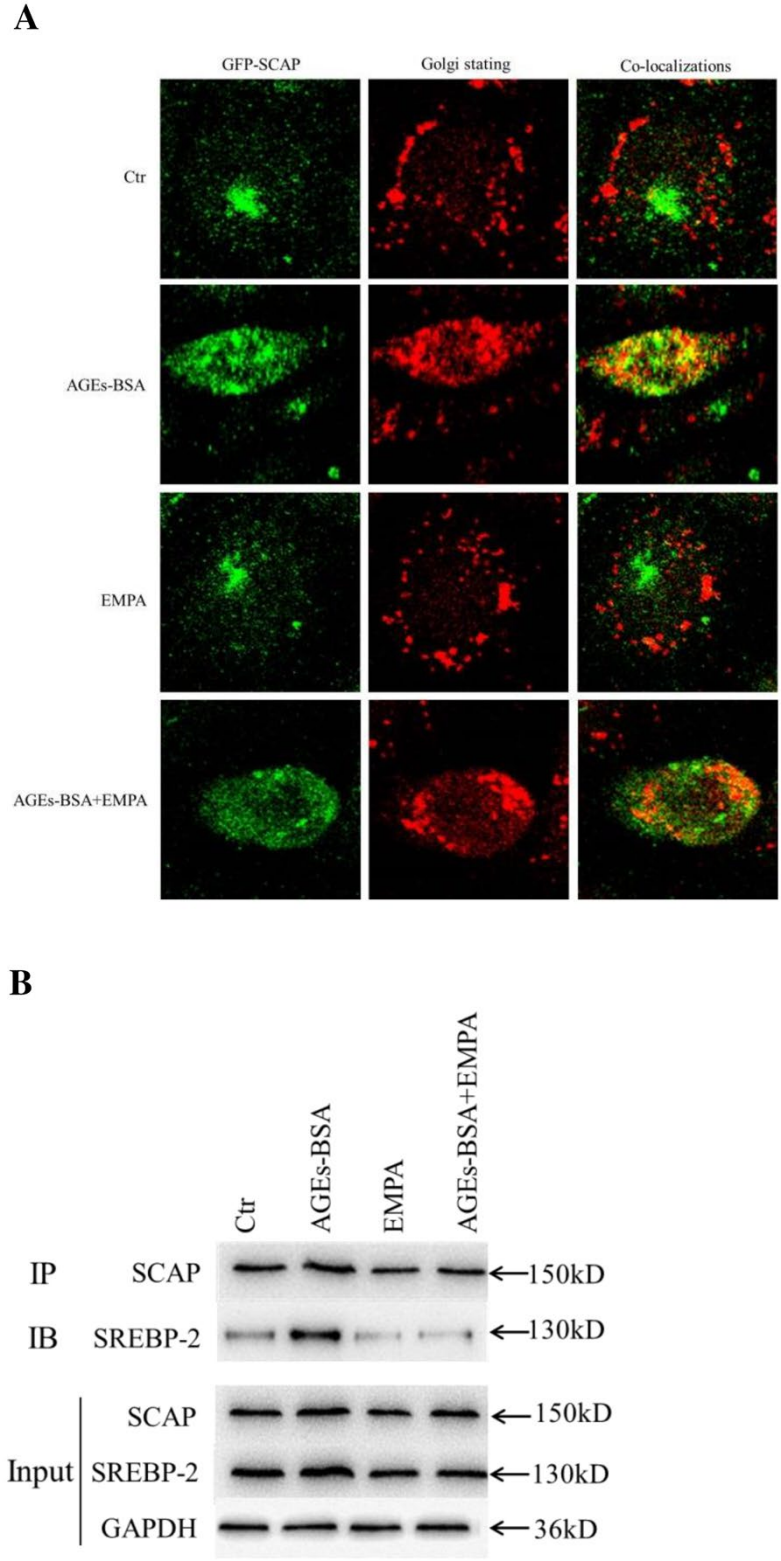
Effect of EMPA on SCAP-SREBP-2 complexes in HK-2 cells. The translocation of GFP-SCAP from the ER to the Golgi was investigated using confocal microscopy after staining with anti-Golgi antibody. EMPA inhibited the translocation of GFP-SCAP from the ER to the Golgi in HK-2 cells (**A**). The interaction between SCAP and SREBP-2 proteins was investigated using Co-IP. AGEs-BSA increased the interactions of SCAP and SREBP-2 in HK-2 cells, whereas this could be inhibited by EMPA (**B**)

We also performed confocal microscopy for observing the ER-to-Golgi translocation of SCAP within the HK-2 cells. It was discovered that AGEs-BSA treatment significantly increased SCAP accumulation within Golgi, but EMPA exposure markedly reduced its accumulation (Fig. 5A). Besides, as revealed by co-IP analysis, SCAP-SREBP-2 complexes were amplified within AGEs-BSA-exposed HK-2 cells, whereas EMPA treatment reduced the SCAP-SREBP-2 interaction (Fig. 5B). Based on the above findings, AGE may contribute to the ER-to-Golgi translocation of SCAP-SREBP-2 complexes through increasing SCAP/SREBP-2 levels, accelerating SREBP-2 hydrolysis. Additionally, nuclear transport of nSREBP-2 fragments was observed, which then bound to LDLr and HMGCoAR promoters to promote their transcription and subsequent translation, inducing cholesterol production and absorption. Interestingly, EMPA reduced SCAP/SREBP-2 expression, diminished SCAP-SREBP-2 protein complex transport, reduced LDLr and HMGCoAR expression, and eventually decreased cholesterol production and absorption.

### Role of EMPA in regulating RAGE level

**Fig. 6:**
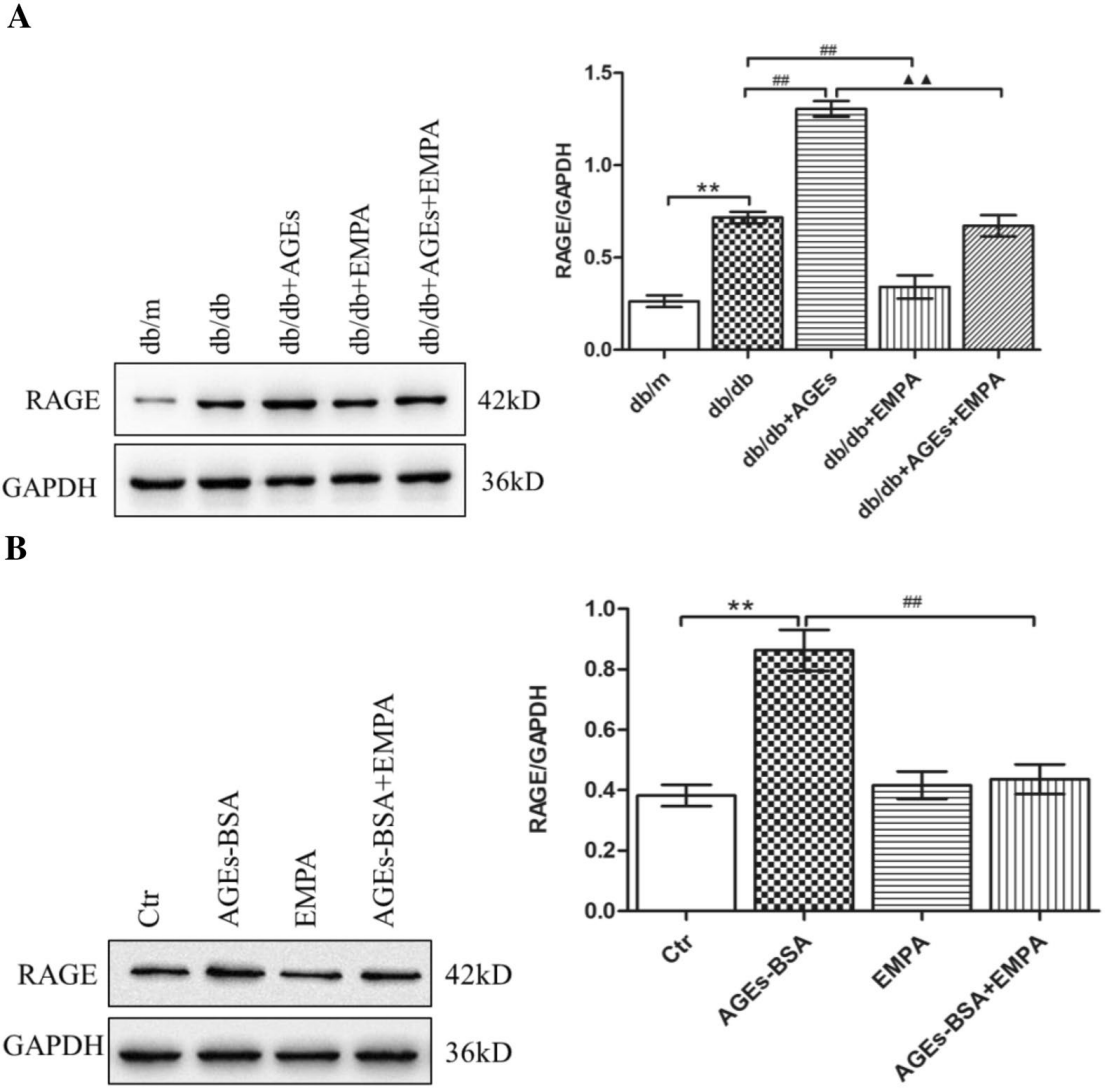
Effects of EMPA on RAGE expression. The protein expression of RAGE in the kidneys of mice (**A**) and HK-2 cells (**B**) was determined by Western blotting. ImageJ was used to quantify the relative levels of proteins. GAPDH was used as an internal control. The values are expressed as the mean ± S.E.M. of 5 independent experiments. **P < 0.01 versus the db/m group or the control cells (Ctr); ^##^*P* < 0.01 versus db/db group or AGEs-BSA treated cells; ^▲▲^*P* < 0.01 versus the AGEs + db/db group

Some specific RAGE exists on the renal tubular surface. AGE binds to RAGE, activates the intracellular signaling pathways, and generates adverse biological effects during DKD. Thus, we evaluated both the in vivo and in vitro RAGE levels. We found that RAGE level was high within db/db mouse kidneys, especially those fed with the AGE-rich diet, relative to db/m mice (Fig. 6A). Similar observations were found in HK-2 cells (Fig. 6B). EMPA exposure decreased the RAGE levels within db/db mice fed with either basal or an AGE-rich diets; similar results could be found within HK-2 cells (Fig. 6A, B).

## Discussion

DKD accounts for over 30% of all CKD cases progressing into end-stage renal disease, which is becoming the major health burden due to the high global prevalence of diabetes. Apart from hyperglycemia, inflammatory factors, hypertension, and renal ectopic lipid deposition are also recognized as the risk factors for DKD. Therefore, it is important to explore the etiology and mechanism of diabetic renal lipid deposition to develop better prevention and treatment strategies. In the earlier study, we reported that excessive consumption of AGEs could disturb intracellular cholesterol negative feedback modulation and induce renal lipid accumulation in db/db mice [11]. However, the relationship between AGEs and renal lipid content has never been reported in diabetic patients. Renal biopsy has proven that there is lipid deposition in the kidneys of patients with T2DM [25]. But due to the invasive procedure involved in renal biopsy, its clinical application is limited. Only a small number of patients, either undergoing kidney surgery or having a renal disease of unknown etiology, will consent to renal tissue biopsy. Therefore, non-invasive renal fat detection needs to be further studied, developed, and popularized. Recently, some studies have reported that MRI is a useful and accurate tool to detect human renal fat content [26, 27]. Additionally, it has been reported that the RFF in patients with T2DM, detected by Dixon MRI, increases significantly compared to the non-diabetic cases [27, 28]. Consistent with the above findings, we show that RFF in T2DM cases increases markedly relative to non-diabetic cases. Furthermore, RFF was significantly correlated with serum AGEs levels, and this correlation between RFF and AGEs was statistically significant even after adjustments for TC, TG, BMI, SCR, FBG, and HbA1c. The previous basic research on AGEs-induced renal lipid deposition might explain the above clinical phenomenon [11]. The results of this study are encouraging since it is the first study to report that EMPA significantly reduces RFF in patients with T2DM. Though it is unclear whether this was related to the reduced levels of AGEs after EMPA treatment, further detailed in vivo and in vitro studies should provide more answers.

The data obtained from the experiments showed the increased amount of intracellular cholesterol and lipid droplets in kidneys, particularly in renal tubules. We also identified the renal functional impairment and pathological changes, especially in diabetic mice fed with an AGE-rich diet. But, EMPA markedly improved these pathological conditions. AGEs induced lipid droplet accumulation was inhibited in EMPA-exposed HK-2 cells. To understand the role of EMPA in tubular lipid accumulation, we analyzed how EMPA affected cholesterol feedback modulation within db/db mouse kidneys as well as HK-2 cells. We found that LDLr, SREBP-2, HMGCoAR, SCAP, and nSREBP-2 were up-regulated in kidneys of diabetic mice, particularly in AGE-rich diet-fed diabetic mice. Similar findings were observed within AGEs-exposed HK-2 cells. Therefore, EMPA decreases renal proteins in diabetic mice, as well as mice and HK-2 cells raised with an AGE-rich diet. Moreover, AGE-BSA exposure stimulated SCAP-SREBP-2 complex formation while increasing their transport to Golgi apparatus but was suppressed via EMPA within HK-2 cells. These observations suggest that AGEs increase SCAP/SREBP-2 levels and promote SCAP-SREBP-2 complex formation. Subsequently, ER-to-Golgi transport of complexes induced SREBP-2 hydrolysis, nuclear import of nSREBP-2 also promoted LDLr/HMGCoAR transcription and translation, elevated cholesterol production and absorption within renal tubules, and finally caused the formation of tubular foam cells. Incidentally, EMPA decreased SCAP/SREBP-2 expression, reduced ER-to-Golgi transport of SCAP-SREBP-2 complexes, reduced SREBP-2 targeted gene transcription and decreased cholesterol production and absorption within renal tubules.

Our previous studies have shown that anti-RAGE could suppress SCAP/SREBP-2 levels, and reduce lipid deposition in HK-2 cells [10]. Based on this, we further studied the impact of EMPA on the AGEs-RAGE axis. The renal RAGE protein expression and serum AGEs contents were elevated in db/db mice, especially in AGE-rich diet-fed db/db mice, relative to db/m mice. EMPA significantly suppressed renal RAGE expression and serum AGEs content within basal and AGE-rich diet-fed db/db mice. Additionally, EMPA also decreased RAGE protein expression in AGE exposed HK-2 cells. Consequently, EMPA decreased renal lipid deposition by reducing AGEs synthesis and suppressing AGEs-RAGE signal transduction, thereby inhibiting the SCAP-SREBP-2-HMGCoAR/LDLr pathway.

Certain limitations should be noted in the present work. Firstly, the sample size in the present clinical work was small, which could have affected the accuracy of our result. The cause-effect relation should be determined on large-scale longitudinal studies. Secondly, EMPA improved renal function and pathological changes under in-vivo conditions. It is possibly associated with reduced renal lipid accumulation. However, it can’t eliminate the impact of blood glucose decreased by EMPA. Thirdly, although EMPA reduced renal cholesterol levels, it made no difference to blood TC level, and it might be associated with EMPA decreasing blood volume, thereby increasing TC concentration. Additionally, EMPA may directly affect the local lipid metabolism of the kidney, and whether it affects blood lipids still requires large clinical studies.

Collectively, this work indicates that AGEs-RAGE interaction can up-regulate SCAP/SREBP-2 expression within renal tubular cells, thereby increasing SCAP-SREBP-2 complex formation and accelerating its transport to Golgi, in which SREBP-2 is hydrolyzed. The nSREBP-2 enters the cell nucleus to increase LDLr and HMGCoAR expression; promotes cholesterol production and absorption. But EMPA treatment decreases AGEs production and suppresses activation of AGEs-RAGE signal pathway, thereby inhibiting the SCAP-SREBP-2-LDLr/HMACoAR pathway and mitigating renal lipid deposition.

## Data Availability

The datasets analyzed in this study are available from the corresponding author (sunhong_611@126.com) upon reasonable request.
